# Secretory phospholipase A2-X (*Pla2g10*) is a novel progesterone receptor target gene exclusively induced in uterine luminal epithelium for uterine receptivity in mice

**DOI:** 10.1186/s13578-020-00495-z

**Published:** 2020-11-19

**Authors:** Hee Kyoung Park, So Hee Park, Miji Lee, Gyeong Ryeong Kim, Mira Park, Seung Chel Yang, Yeon Sun Kim, Hyunjung J. Lim, Hye-Ryun Kim, Haengseok Song

**Affiliations:** 1 Department of Biomedical Science, CHA University, Gyeonggi-do, Seongnam-si, 13488 Republic of Korea; 2 Department of Veterinary Medicine, Konkuk University, Seoul, 05029 Republic of Korea; 3 Present Address: Division of Endocrinology and Metabolism, Department of Internal Medicine, Collage of Medicine, Gil Medical Center, Gachon University, Incheon, 21565 Republic of Korea

**Keywords:** *Pla2g10*, Progesterone receptor, Uterine epithelium, Uterine receptivity, Embryo implantation

## Abstract

**Background:**

Aberration of estrogen (E_2_) and/or progesterone (P_4_) signaling pathways affects expression of their target genes, which may lead to failure of embryo implantation and following pregnancy. Although many target genes of progesterone receptors (PRs) have been identified in uterine stroma, only a few PR targets have been reported in the epithelium. Secretory phospholipase A_2_-(PLA_2_)-X, a member of the PLA_2_ family that releases arachidonic acids for the synthesis of prostaglandins that are important for embryo implantation, is dysregulated in the endometrium of patients suffering from repeated implantation failure. However, it is not clear whether sPLA_2_-X is directly regulated by ovarian steroid hormones for embryo implantation in the uterus.

**Result:**

P_4_ induced the *Pla2g10* encoding of secretory PLA_2_-X in the apical region of uterine LE of ovariectomized mice via PR in both time- and dose-dependent manners, whereas E_2_ significantly inhibited it. This finding is consistent with the higher expression of *Pla2g10* at the diestrus stage, when P_4_ is elevated during the estrous cycle, and at P_4_-treated delayed implantation. The level of *Pla2g10* on day 4 of pregnancy (day 4) was dramatically decreased on day 5, when PRs are absent in the LE. Luciferase assays of mutagenesis in uterine epithelial cells demonstrated that four putative PR response elements in a *Pla2g10* promoter region are transcriptionally active for *Pla2g10*. Intrauterine delivery of small interfering RNA for *Pla2g10* on day 3 significantly reduced the number of implantation sites, reinforcing the critical function(s) of *Pla2g10* for uterine receptivity in mice.

**Conclusions:**

*Pla2g10* is a novel PR target gene whose expression is exclusively localized in the apical region of the uterine LE for uterine receptivity for embryo implantation in mice.

## Background

It is well understood that prostaglandins (PGs) are critical for sequential events of female reproduction from ovulation to parturition [1–3]. PGs are generated from arachidonic acid (AA) by phospholipase A_2_s (PLA_2_s) followed by cyclooxygenases. PLA_2_ enzymes are classified into two groups, cytosolic and secretory. Cytosolic PLA_2_s (cPLA_2_s), which are regulated by Ca^2+^-dependent translocation and phosphorylation, have a preference for AA in membrane phospholipids and play an essential role in agonist-induced AA release. The cPLA_2_α-derived AA is important for the PG synthesis that is required for on-time implantation [1]. Several secretory PLA_2_s (sPLA_2_s), including groups IIA, III, V, and X, are likely to be involved in AA release and subsequent eicosanoid production during inflammatory conditions [4]. Several sPLA_2_s, as well as cPLA_2_sα, are spatiotemporally induced in mouse uterus for uterine receptivity [1]. It was previously reported that *PLA2G10* encoding of sPLA_2_-X is dysregulated in the endometrium of patients with repeated implantation failure (RIF) [5]. However, detailed mechanism(s) by which ovarian steroid hormones regulate expression of *Pla2g10* in the uterus remain unanswered.

Ovarian steroid hormones, estrogen (E_2_) and progesterone (P_4_), orchestrate dynamic changes in the uterus during reproductive cycles [6–8]. These hormones act on uterine physiology mainly via their own nuclear receptors; namely, estrogen receptors and progesterone receptors (PRs), respectively [9, 10]. Sophisticated actions of these hormones on major uterine cell types, including various immune cells, are prerequisites for changing the uterine environment from the pre-receptive to the receptive phase for successful embryo implantation [11–13]. Desynchronized actions of these hormones may provide various causes of RIF. P_4_ play critical roles for the establishment and maintenance of pregnancy by not only its endocrine but also immunological effects [14–16]. P_4_–PR transcriptional network along with estrogen signaling promotes spatiotemporal regulation of various target genes for achieving uterine receptivity in the uterus [17]. Whereas most of the PR target genes are expressed in stromal cells, several genes including Amphiregulin (*Areg*), Indian hedgehog (*Ihh*), Calcitonin (*Ct*), GATA binding protein 2 (*Gata2*), and sex-determining region Y-related high-mobility group box 17 (*Sox17*) have been identified in the uterine epithelium to date [18–22]. Here we demonstrate that *Pla2g10*, one of dysregulated genes in the endometrium of patients with RIF, is a novel PR target gene that is exclusively induced in uterine luminal epithelium (LE) for uterine receptivity for embryo implantation in mice.

## Results

### ***PLA2G10*** dysregulated in human endometrium of patients with RIF is regulated by P_4_

**Fig. 1:**
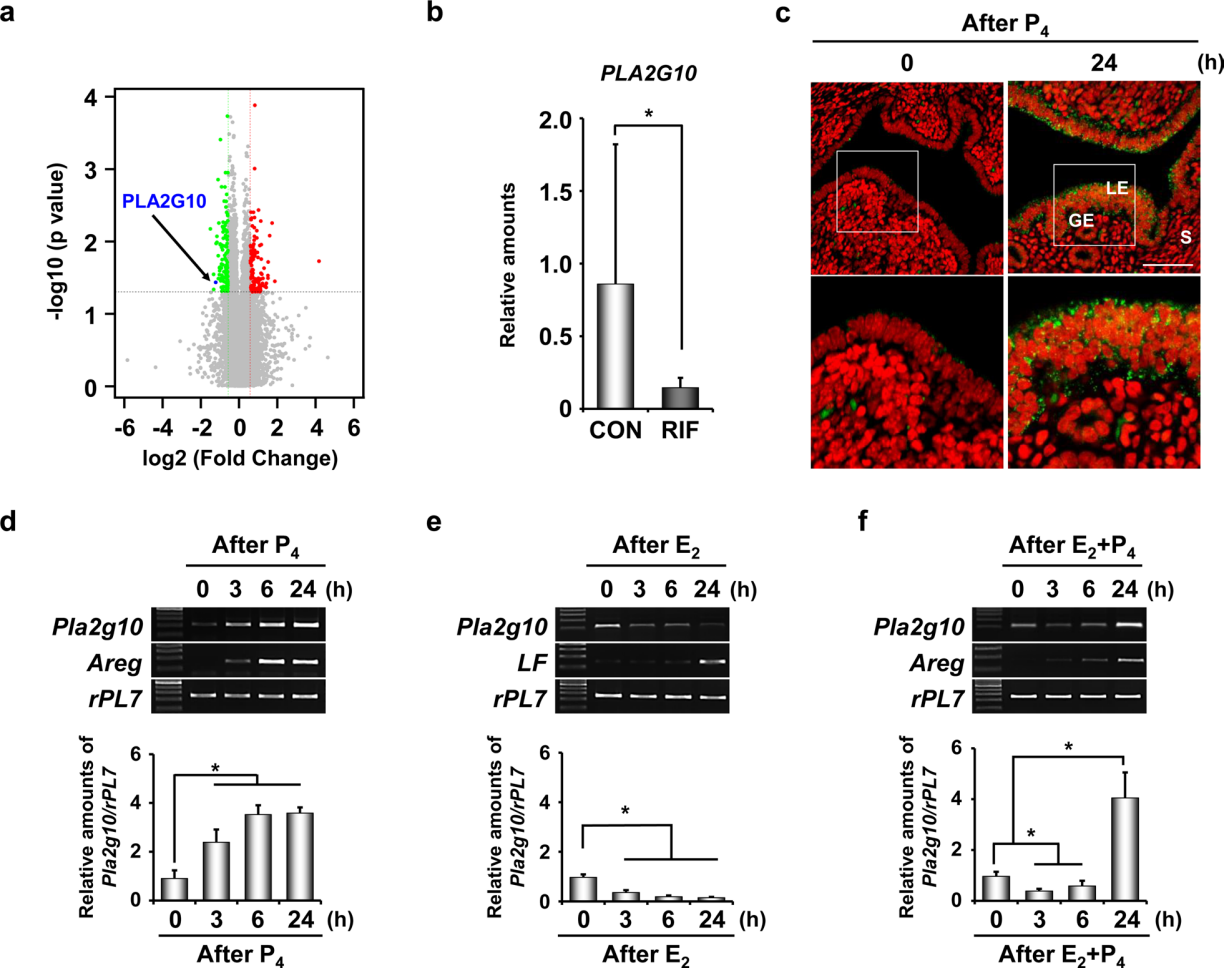
*Pla2g10* expression is positively regulated by P_4_ in the uterus. **a** A volcano plot to compare expression profiles of the mid-luteal phase endometria from the healthy women (CON, n = 6) vs patients with RIF (n = 9). *PLA2G10* is visualized as one of downregulated genes in the endometria of patients with RIF. **b** Real-time RT-PCR analysis for relative mRNA levels of *PLA2G10* between endometria of CON and patients with RIF in mid-luteal phase. **c** Immunofluorescence of PLA2G10 24 h after P_4_ treatment in uteri of OVX mice. The bottom panels show higher magnification images of the boxed area. Green and red colors indicate the presence of PLA2G10 and nuclei, respectively. LE, luminal epithelium; GE, glandular epithelium; S, stroma. Scale bar: 50 µm. **d–f** RT-PCR and real-time RT-PCR results to analyze relative levels of *Pla2g10* mRNA by **d** P_4_ (2 mg/mouse), **e** E_2_ (200 ng/mouse), and **f** E_2_ + P_4_ treatment in uteri of OVX mice at different time points (n = 4–5 mice for each time point). Expression levels of *Areg* and *LF* mRNAs were also evaluated to validate appropriate P_4_ and E_2_ hormone responses in OVX mice used in this experiment, respectively. *p < 0.05

Previously, we demonstrated that a group of genes, including *PLA2G10*, is dysregulated in the endometrium of patients with RIF [5]. Volcano plots and real-time reverse transcriptase-polymerase chain reaction (RT-PCR) for endometrial samples demonstrate that *PLA2G10* mRNAs were significantly down-regulated in the endometrium of patients with RIF at mid-luteal phase (Fig. 1a, b). To further investigate the underlying mechanism of dysregulated expression of *PLA2G10* in the endometrium of patients with RIF, we examined steroid hormonal regulation of *Pla2g10* in mouse uterus. Because P_4_ is an essential steroid hormone to prepare embryo implantation in the uterus, it was first examined whether *Pla2g10* expression is regulated in the uterus by P_4_ using an ovariectomized (OVX) mouse model. P_4_ was given to OVX mice whose uteri were collected at different time points (0, 3, 6, and 24 h) after hormone treatment (Fig. 1c, d). The RT-PCR and real-time RT-PCR results demonstrate that *Pla2g10* expression was gradually increased by P_4_ in a time-dependent manner, with the highest level at 24 h (Fig. 1d). Immunofluorescence staining shows that PLA2G10 is mainly localized in the apical region of LE cells in mouse uterus (Fig. 1c). These results suggest that *Pla2g10* may be a novel P_4_ target gene that is exclusively induced in the LE of the uterus.

### E_2_ inhibits both basal and P_4_-dependent expression of *Pla2g10* in mouse uterus in a time-dependent manner

To investigate the effects of E_2_ on *Pla2g10* expression in mouse uterus, E_2_ with or without P_4_ was given to OVX mice whose uteri were collected at different time points after hormone treatment(s). A single injection of E_2_ significantly reduced basal levels of *Pla2g10* mRNAs in mouse uterus of OVX mice (Fig. 1e). Furthermore, E_2_ suppressed P_4_-dependent induction of *Pla2g10* at 3 and 6 h after hormone treatments, whereas the inhibitory action was no longer effective at 24 h (Fig. 1f). These results suggest that E_2_ has inhibitory actions on basal and P_4_-dependent expression of *Pla2g10* in mouse uterus.

### P_4_ regulates ***Pla2g10*** induction via its nuclear PR in a dose-dependent manner

**Fig. 2:**
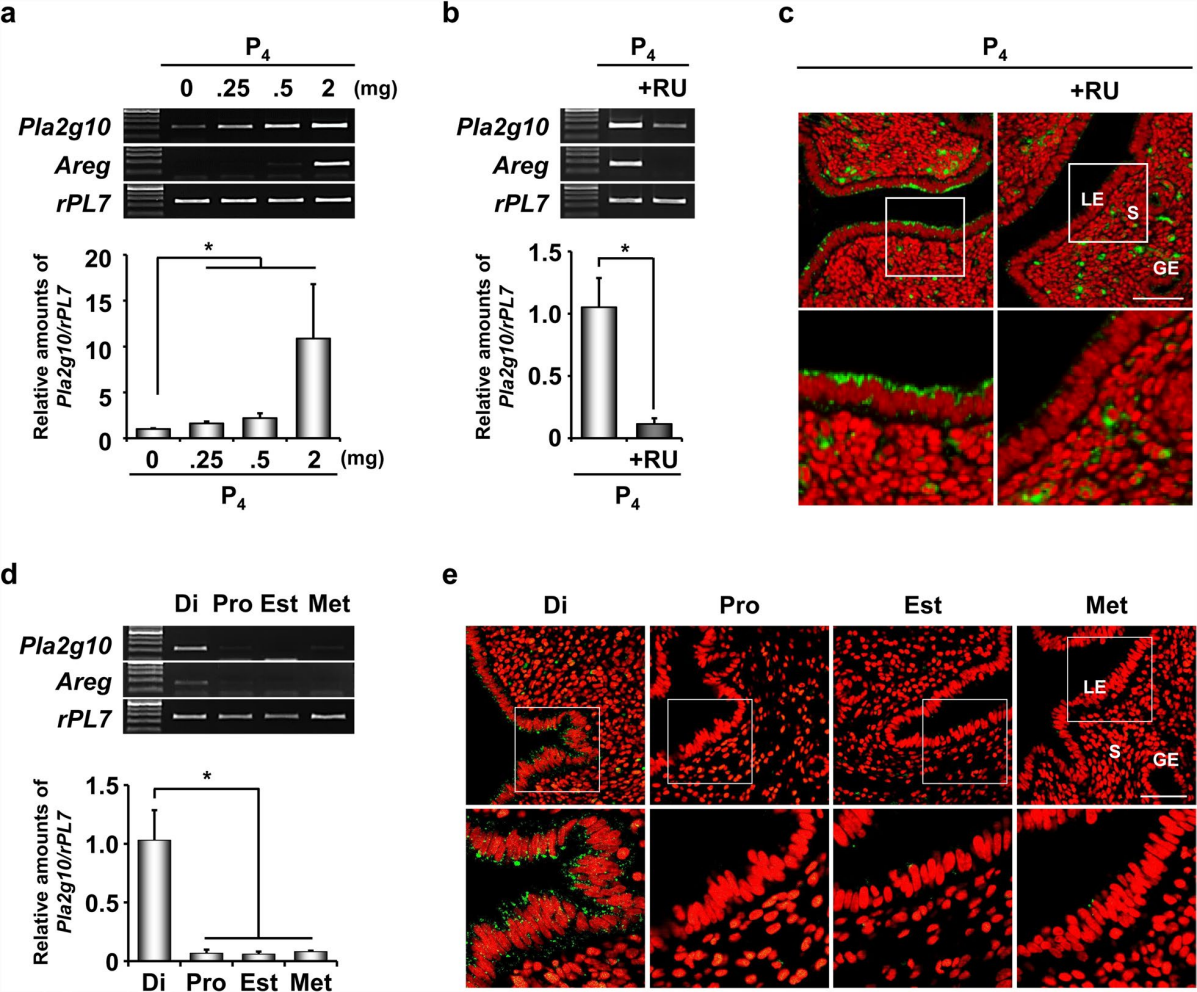
P_4_-dependent induction of *Pla2g10* is mediated via its nuclear receptor, PR, during estrous cycle in the uterus. **a** Analyses of expression levels of *Pla2g10* mRNAs in mouse uterus exposed to various concentrations of P_4_ (0.25–2 mg). **b-c** Evaluation of the inhibitory action of PR antagonist, RU-486 (1 mg/mouse) on P_4_ (2 mg/mouse)-induced *Pla2g10* expression in uteri of OVX mice (n = 5 per each group) by **b** RT-PCR and real-time RT-PCR and **c** immunofluorescence. The bottom panels show higher magnification images of the boxed area. **d** Analyses of expression levels of *Pla2g10* mRNAs during estrous cycle. **e** Immunofluorescence of PLA2G10 in the uterus during estrous cycle. The bottom panels show higher magnification images of the boxed area. Green and red colors indicate the presence of PLA2G10 and nuclei, respectively. *RU* RU-486, *LE* luminal epithelium, *GE* glandular epithelium, *S* stroma, *Di* diestrus, *Pro* proestrus, *Est* estrus, *Met* metestrus. Scale bar: 50 µm. *p < 0.05

To investigate whether *Pla2g10* expression is regulated by P_4_ in a dose-dependent manner, various concentrations (0.25–2 mg) of P_4_ were given to OVX mice and *Pla2g10* expression was evaluated 24 h after P_4_ injection. RT-PCR and real-time RT-PCR analyses show *Pla2g10* induction by P_4_ in a dose-dependent manner, with a peak level in uterine samples with 2 mg (Fig. 2a). To determine whether P_4_-induced *Pla2g10* expression is mediated via nuclear PRs in mouse uterus, OVX mice were pretreated with a PR antagonist RU-486 30 min before P_4_ injection. RU-486 pretreatment significantly abrogated P_4_-dependent induction of *Pla2g10* as well as *Areg*, a known P_4_ target gene expressed in the LE of mouse uterus. These results suggest that P_4_-dependent induction of *Pla2g10* expression works through nuclear PR in the uterus (Fig. 2b, c).

### Expression of ***Pla2g10*** is elevated in P_4_-dominant diestrus stage during estrous cycle

To further understand P_4_-dependent regulation of *Pla2g10* in the uterus, we examined its expression in the uterus at different stages of the estrous cycle during which the uterus undergoes cyclic hormonal changes. Consistent with hormone-dependent profiles of *Pla2g10* expression, it was notably expressed in the diestrus stage when P_4_ is dominant, but not in the proestrus and estrus stages when levels of E_2_ are high (Fig. 2d, e). Accordingly, PLA2G10 was mainly detected in the LE of mouse uterus in the diestrus stage (Fig. 2e).

### *Pla2g10* expression coincides with PR in the LE for uterine receptivity for embryo implantation

**Fig. 3:**
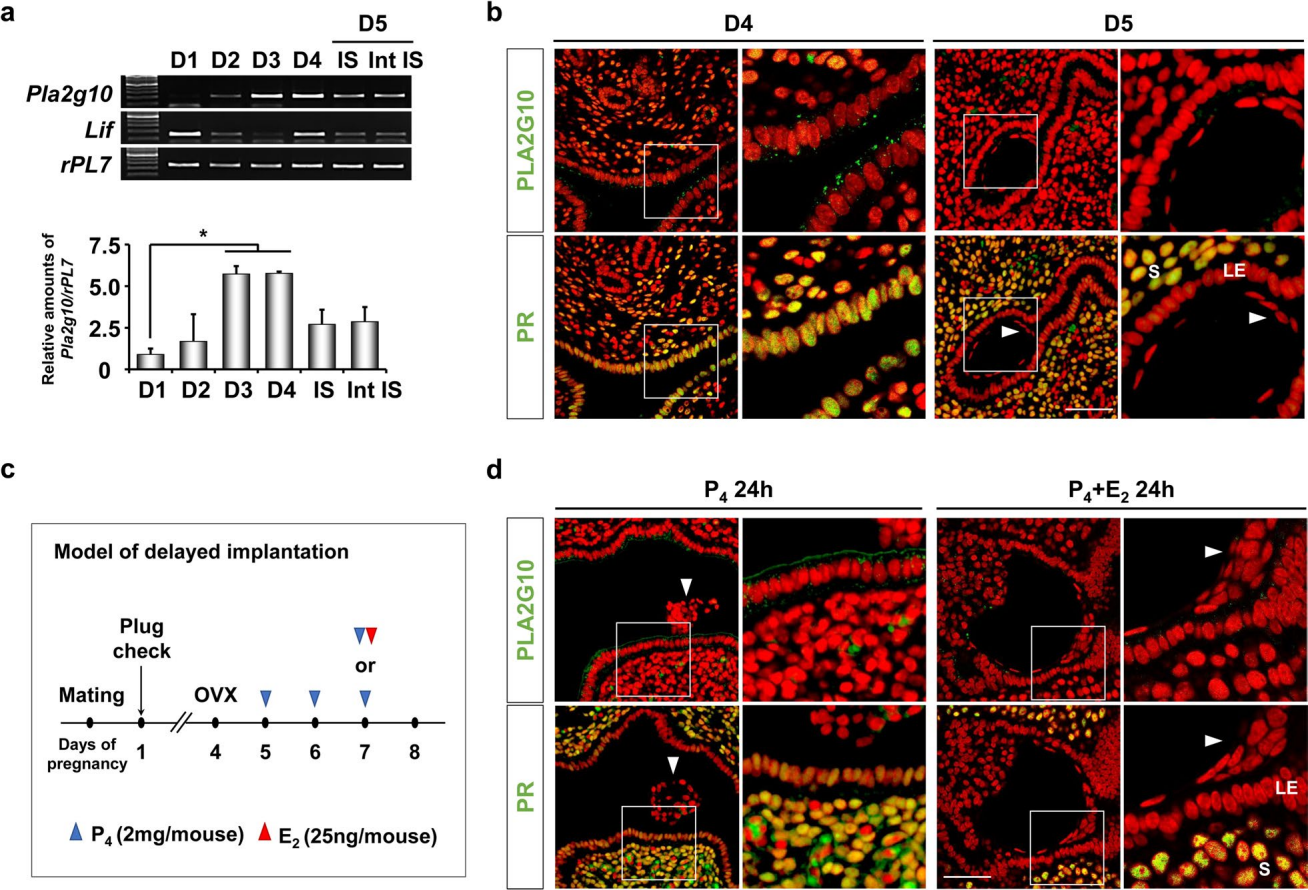
PR-dependent *Pla2g10* expression in the uterus during early pregnancy and a delayed implantation model. **a** RT-PCR and real-time RT-PCR analyses to monitor the relative levels of *Pla2g10* mRNAs in the uterus on days 1 to 5 of pregnancy (D1–D5). *IS* implantation site, *int IS* inter IS. **b** Immunofluorescence of PLA2G10 and PR in uteri on D4 and D5. **c** A schematic diagram to show the experimental schedule to experimentally-induced delayed implantation. **d** Immunofluorescence of PLA2G10 and PR in uteri on the delayed implantation model. The right panels show higher magnification images of the boxed area. Green and red colors indicate the presence of PLA2G10 or PR and nuclei, respectively. Arrowheads indicate the location of implanted blastocyst. *LE* luminal epithelium, *S* stroma. Scale bar: 50 µm

During early pregnancy in mice, the uterus is influenced by P_4_ from newly formed corpus lutea from day 3 of pregnancy (day 3) onwards [6]. Thus, we examined expression patterns of *Pla2g10* in mouse uterus during early pregnancy. *Pla2g10* was highly expressed on days 3 and 4, whereas it remained at basal levels on days 1 and 2 when E_2_ was dominant (Fig. 3a). Interestingly, P_4_-dependent expression of *Pla2g10* was significantly reduced in both implantation site (IS) and inter-IS on day 5 (Fig. 3b). Similar observation was made in the uterus on days 4 and 5 of pseudopregnancy (data not shown). Considering that P_4_ levels are similarly maintained on days 4 and 5 [23, 24], downregulation of PLA2G10 in the LE on day 5 may be associated with loss of PR in this compartment. In fact, it is interesting to observe that PR as well as and PLA2G10 is dramatically reduced in the LE irrespective of the implanting blastocyst on day 5 (Fig. 3b) and day 5 of pseudopregnancy (data not shown).

### ***Pla2g10*** is regulated by P_4_-PR-dependent signaling in mouse uterus during delayed implantation

To further evaluate whether the sudden decrease of the PLA2G10 expression in the LE on day 5 is caused by loss of PR, we used an experimentally-induced delayed implantation model (DIM) in mice (Fig. 3c). PLA2G10 expression was maintained in a P_4_-primed uterus at a state of dormancy (P_4_ 24 h). However, 24 h after termination of delayed implantation with an injection of E_2_ (P_4_ + E_2_ 24 h), it disappeared in the LE in mouse uterus (Fig. 3d). The loss of PLA2G10 in the LE at P_4_ + E_2_ 24 h coincided with loss of PR in the LE during DIM. Taken together, these results suggest that PLA2G10 expression exclusively depends on PR in mouse uterus during early pregnancy and DIM.

### *Pla2g10* promoter has functional PREs

**Fig. 4:**
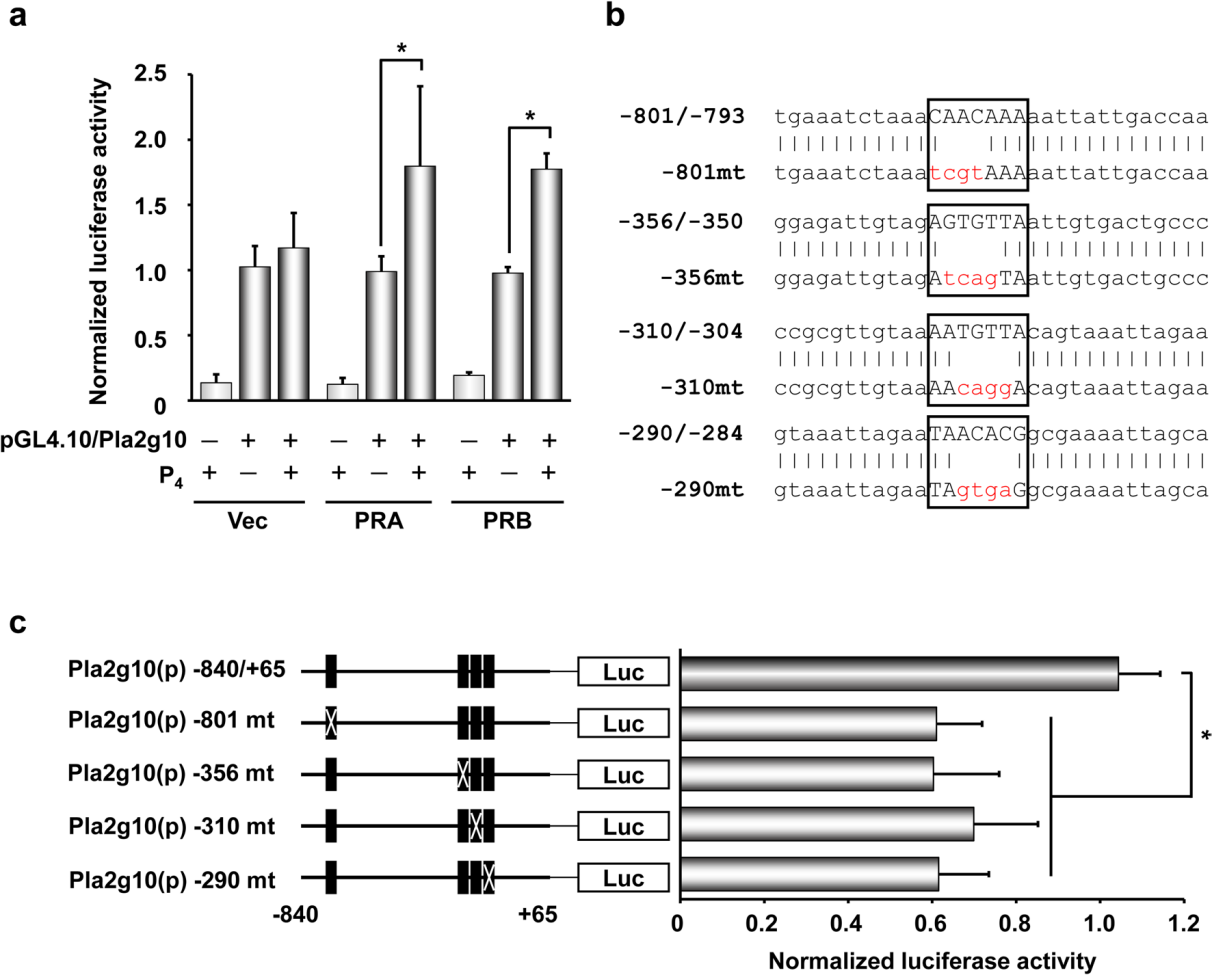
*Pla2g10* promoter has functional PREs in uterine epithelial cells. **a** Luciferase assays for a proximal region of *Pla2g10* promoter in Ishikawa cells where control (Vec), PRA, or PRB vectors were co-transfected with pGL4.10/Pla2g10 luciferase vector under 1 µM P_4_. Vec: empty vector. **b** A schematic cartoon to show *Pla2g10* promoter that contains putative PREs that were mutated by site-directed mutagenesis. Box, PRE sequence. Red letter, mutated sequence. **c** Luciferase assays for *Pla2g10* promoter constructs with a mutation (mt) on each PRE in Ishikawa cells under the same conditions as **a**. The normalized luciferase activities are shown by ratio to renilla activity. Data are presented as the mean ± SD of three independent experiments. *p < 0.05

To further understand the molecular mechanism(s) by which the P_4_-PR signaling pathway regulates *Pla2g10* expression at transcriptional levels, a series of luciferase assays with a proximal promoter region of *Pla2g10* gene containing putative PR response elements (PREs) was performed in Ishikawa cells, human endometrial adenocarcinoma cells. In silico analyses via a PROMO program (http://alggen.lsi.upc.es) suggest that four putative PREs were found in − 840/ + 65 of the *Pla2g10* promoter. These PREs were also validated by ChIP-seq analyses in a previous study [25]. The luciferase activity of the *Pla2g10* promoter was significantly increased when co-transfected with PRA or PRB expression vector along with P_4_ (Fig. 4a). To determine which PRE is functionally critical for PR-dependent *Pla2g1*0 transcription, four putative PREs in the *Pla2g10* proximal promoter region (− 840/ + 65) were mutated (Fig. 4b). All four mutations (mt) at − 801/− 793, − 356/− 350, − 310/− 304, and − 290/− 284 PREs at the *Pla2g10* promoter showed about 40% reduced luciferase activity when co-transfected with PR(s) (Fig. 4c). These results indicate that *Pla2g10* transcription is directly regulated by PR in the uterus.

### In vivo delivery of siRNA to knock-down *Pla2g10* disturbs embryo implantation

**Fig. 5:**
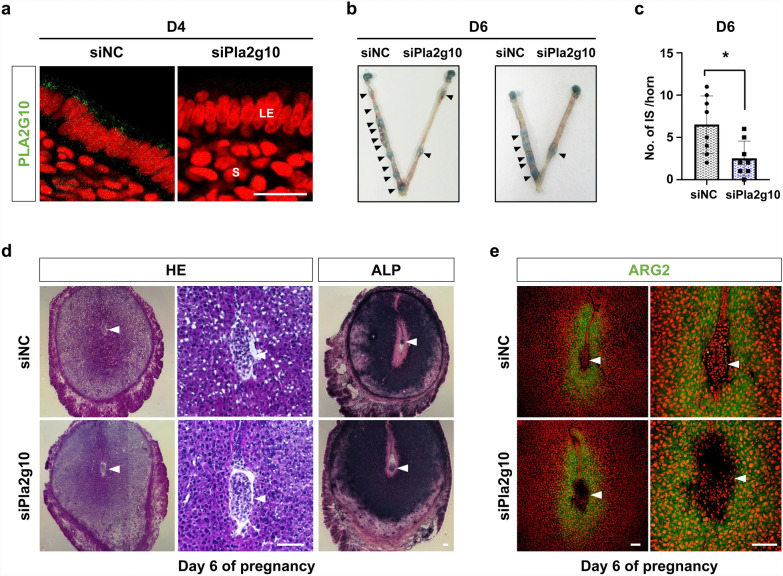
In vivo delivery of siRNAs for *Pla2g10* interferes with embryo implantation. **a** Immunofluorescence analysis represent PLA2G10 in mouse uteri treated with siNC or siPla2g10 (100 pmol per uterine horn). Green and red colors indicate the presence of PLA2G10 and nuclei, respectively. *LE* luminal epithelium, *S* stroma. Scale bar: 20 µm. **b** Representative photograph of a mouse uterus injected with siNC or siPla2g10 with arrowheads indicating implantation sites. **c** Number of implantation site (IS) was counted on day 6 of pregnancy in mouse uteri treated with siNC or siPla2g10 (100 pmol per uterine horn, n = 8 per each group). **d** Photomicrographs of representative uterine sections showing H&E staining and ALP activity on day 6 IS in the uterus with siNC or siPla2g10. Right panels of H&E show the enlarged images of IS. Scale bar: 100 µm. **e** Immunofluorescence staining of ARG2 on day 6 IS in the uterus with siNC or siPla2g10. Arrowheads indicate the location of implanted embryo. Scale bar: 100 µm. *p < 0.05

**Fig. 6:**
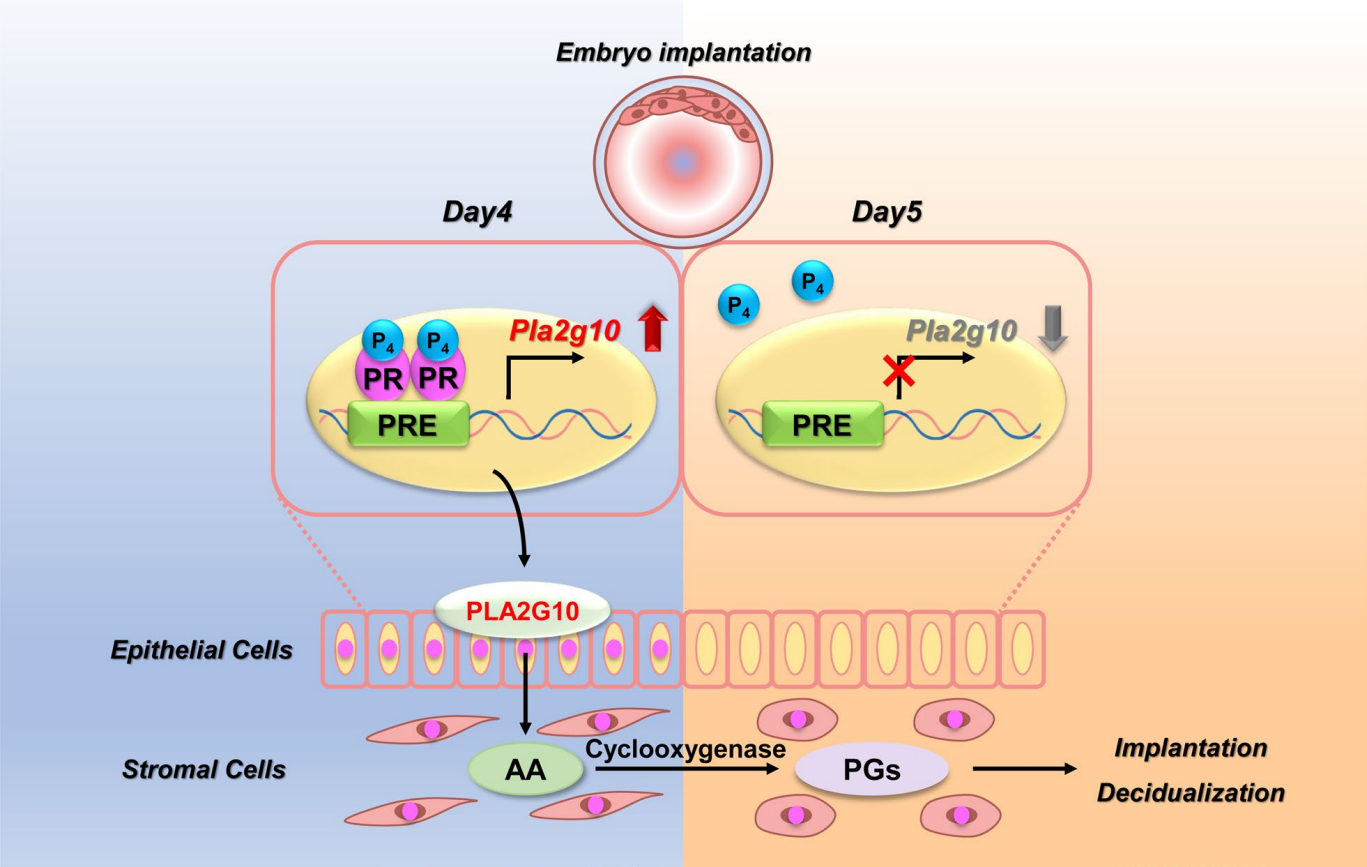
A schematic cartoon to describe the molecular mechanism by which P_4_–PR regulates *Pla2g10* expression for uterine receptivity for embryo implantation in the luminal epithelium in mouse uterus

We next performed in vivo interference of *Pla2g10* to investigate whether *Pla2g10* contributes to embryo implantation in mouse uterus. Intrauterine injection of siRNA against *Pla2g10* (siPla2g10) (100 pmol per uterine horn) on day 3 caused significant knock-down of PLA2G10 expression on day 4 (Fig. 5a). In vivo interference of *Pla2g10* expression with siPla2g10 in mouse uterus significantly decreased the number of IS on day 6 (6.5 vs 2.5) compared to the control horns with negative control siRNA (siNC) (Fig. 5b, c). However, a few embryos successfully implanted in mouse uterus with siPla2g10. Gross histology and ALP staining for IS on day 6 showed that implantation normally occurs in the uterus with siPla2g10 (Fig. 5d). Furthermore, ARG2 localization in the decidualizing stromal cells surrounding the implanted embryo on day 6 was similarly observed between uteri with siPla2g10 and siNC (Fig. 5e), suggesting that the uterine environment could be locally disturbed, but not systemically altered in mouse uterus by siPla2g10. Collectively, these results suggest that P_4_-PR signaling induces expression of *Pla2g10* in LE, which participates in PG biosynthesis critical for uterine receptivity for embryo implantation in mice (Fig. 6).

## Discussion

*Pla2g10* is known as a Ca^2+^-dependent low molecular-weight enzyme (13–18 kDa) that is involved in biosynthesis of PGs, an important lipid mediator for embryo implantation [26, 27]. We previously demonstrated that mice deficient of *Pla2g4a*, a cytosolic form of PLA_2_, have aberrant uterine spacing of embryos and deferred embryo implantation. The deferred implantation and fetal growth restriction in *Pla2g4a* deficient mice were significantly recovered by exogenous PG administration [1]. Subsequent studies have supported this notion that PLA_2_s-derived AA is important for PG synthesis that is crucial for on-time implantation [28–30]. In our previous study, PLA2G10 was identified as a dysregulated gene in microarray analyses in the endometrium of patients with RIF in whom P_4_ signaling could be locally impaired [5]. In fact, *Pla2g10* expression was gradually increased by P_4_ in a dose-dependent manner (Fig. 1). In line with this result, a recent study shows that the role of PLA_2_s in acrosome reaction in vitro depends on P_4_ concentration [31]. In this study, we clearly demonstrate that *Pla2g10* is a novel PR target gene whose expression is exclusively induced in the LE in mouse uterus.

Molecular cross-talks between the blastocyst and the uterus induce growth factors, adhesion molecules, cytokines, and transcription factors to prepare uterine conditions for embryo implantation [6, 32–35]. P_4_–PR-target gene networks are known to have critical functions for embryo implantation [8, 36, 37]. Most of PR target genes, such as *Hoxa10* and *Hand2*, are expressed in stromal cells [8, 17]. Only several PR target genes, such as *Areg*, *Ihh*, *CT*, *Gata2*, and *Sox17* have been identified in the epithelial compartment [18–22]. *Areg* is a well-known PR target gene whose expression is increased in the uterine epithelium in response to P_4_ for uterine receptivity. With the onset of blastocyst attachment late on day 4, *Areg* mRNA accumulated in the LE exclusively at the sites of blastocysts [18], which may compensate for the deficiency of HB-EGF around the time of embryo implantation in the uterus of HB-EGF knockout mice [38]. Very high levels of *Ihh* mRNA are seen in the luminal and glandular epithelia on day 3 for preparing embryo implantation [19]. Consistent with these results, *Pla2g10* is expressed in LE during the early pregnancy (Fig. 3). P_4_ promotes expression of not only *Pla2g10*, a Ca^2+^-dependent enzyme, but also CT in uterine epithelium [20, 39]. Interestingly, CT leads to increased concentration of intracellular Ca^2+^, suggesting that the P_4_–PR signaling, probably via CT induction, could regulate *Pla2g10* expression and functional activities for uterine receptivity for embryo implantation [20, 40]. *Gata2*, a P_4_ target gene, is colocalized in the uterine epithelium during early pregnancy with PR, and promotes expression of Pgr gene but also regulates downstream progesterone responsive genes, such as *Sox17*, in conjunction with the PR [21, 22, 41]. We also found three putative GATA binding sites in nearby − 801/− 793 PRE of the *Pla2g10* promoter (data not shown). Thus, it is suggested that *Pla2g10* expression could be regulated in the uterine epithelium via PR–GATA2 dependent manner.

It is well-known that stromal PR is the major regulator of the expression of P_4_ target genes and the ability of P_4_ to inhibit E_2_-induced epithelial cell proliferation [42]. However, a recent study shows that epithelial PR acts to inhibit E_2_-induced epithelial proliferation and is essential for uterine development and function, suggesting the importance of epithelial PR for embryo implantation [43]. During early pregnancy, PR is transiently expressed in the epithelium just prior to embryo implantation [43, 44]. After embryo implantation occurs, PR expression in the epithelium rapidly decreases [44] whereas its expression in uterine stroma increases and persists throughout decidualization in mice [45]. Loss of PR expression in the uterine epithelium is crucial for luminal closure for embryo implantation [46]. A previous report suggests that E_2_ down-regulates PR in uterine epithelium through paracrine actions mediated by stromal ERα [47]. This could support the notion that a rapid decrease of P_4_-dependent *Pla2g10* expression in the epithelium in mouse uterus may be caused by reduction of epithelial PR (Fig. 3b). In fact, we found that expression of P_4_-dependent *Pla2g10* was suppressed by E_2_ in uteri of OVX mice (Fig. 1f). This notion is supported by the results that *Pla2g10* promoter has functional PREs (Fig. 4c) and expression of PLA2G10 is synchronized with that of PR in epithelial cells during early pregnancy (Fig. 3a, b). Although *Areg* is specifically induced in uterine epithelium surrounding the implanting blastocyst on day 5, *Pla2g10* is not influenced by the presence of implanting blastocyst (Fig. 3b, d). This suggests that the molecular mechanism by which P_4_–PR signaling regulates *Pla2g10* expression seems to be different from other PR target genes expressed in the epithelium in the mouse uterus during embryo implantation. Intrauterine delivery of siRNA has been performed to elucidate the function of genes on embryo implantation in mice [48–50]. In general, the in vivo action of delivered siRNAs partially inhibits expression levels of target genes and reduces the number of IS at the time of embryo implantation. Figure 5 shows similar results that intrauterine delivery of siRNA for *Pla2g10* inhibited PLA2G10 expression in LE on day 4 and reduced the number of IS on day 6.

## Conclusion

Collectively, this is the first report that *Pla2g10* is a novel P_4_-PR target gene that is exclusively induced in LE to prepare uterine receptivity for embryo implantation in mice (Fig. 6). Further studies are needed to comprehensively understand molecular regulation of steroid hormone receptors on transcriptional activity of the *Pla2g10* promoter.

## Methods

### Animals

All animals were maintained and handled according to the policies approved by CHA University Institutional Animal Care and Use Committee (IACUC, approval number 170002). Eight-week-old adult ICR mice were provided by Orient Bio, Inc (Gapyeong, Gyeonggi, Korea).

### Hormone treatments

To examine the actions of ovarian steroid hormones on expression of *Pla2g10*, adult female mice were OVX, rested for 14 days, and then appropriately treated with steroid hormones for each experiment performed in this study. Mice were sacrificed and their uterine horns were collected for real-time RT-PCR and/or immunofluorescence after ovarian steroid hormone treatment.

To investigate time-dependent actions of P_4_ (Sigma-Aldrich, USA) and E_2_ (17β-estradiol, Sigma-Aldrich) on the expression of *Pla2g10* in mouse uterus, adult OVX mice were subcutaneously injected with P_4_ (2 mg/mouse) or P_4_ + E_2_ (200 ng/mouse) and sacrificed at various time points (0, 3, 6, and 24 h) after injection. To examine the dose-dependent induction of *Pla2g10* by P_4_, mice were given a single injection of vehicle (sesame oil, 0.1 ml/mouse) or P_4_ at various concentrations (0.25–2 mg). To analyze whether P_4_ works through a nuclear PR for *Pla2g10* expression in mouse uterus, adult OVX mice were pretreated with the PR antagonist RU-486 (1 mg/mouse, Sigma-Aldrich), 30 min before P_4_ (2 mg/mouse) injection and then sacrificed 24 h later.

### Preparation of uterine samples during early pregnancy

Uterine samples during early pregnancy were prepared as previously described [35]. Briefly, 8- to 10-week-old female mice were housed with proven fertile males for pregnancy. The next morning when the vaginal plug was found was considered as day 1. Pregnant mice were sacrificed on various days of pregnancy, and their uteri were collected for real-time RT-PCR and/or immunofluorescence. IS in the morning (0900 h) of day 5 and 6 were visualized by intravenous injection (0.1 ml/mouse) of Chicago sky blue 6B solution (1% in saline, Sigma-Aldrich). The IS were demarcated by discrete blue bands along the uterus. IS on day 6 were collected and immediately frozen in liquid nitrogen for frozen section to perform histological analyses including immunofluorescence staining and alkaline phosphatase (ALP) activity assay.

To induce an experimentally-induced delayed implantation model in mice, pregnant ICR female mice were OVX at the morning of day 4 and given P_4_ (2 mg/mouse) from day 5 to 7 as described previously [51]. To activate dormant blastocysts and initiate implantation, P_4_-primed delayed implanting pregnant mice were injected with E_2_ (25 ng/mouse) on day 7. Mice were sacrificed 24 h after the last hormone injection, and IS were visualized using Chicago sky blue 6B solution.

### RNA extraction, RT-PCR, and real-time RT-PCR

The experiment was performed as previously described [35]. Briefly, uteri (3–5 mice per each group) were collected and immediately frozen in liquid nitrogen. Then, total RNA was extracted individually using Trizol Reagent (Ambion, USA) according to manufacturer’s protocols. cDNA was synthesized from total RNA using M-MLV reverse transcriptase (Promega, USA) with random primers and oligo dT. Synthesized cDNA was utilized for PCR with specific primers at optimized cycles. Real-time RT-PCR was performed by monitoring real-time increases in the fluorescence of SYBR Green dye. Real-time RT-PCR was performed using the Realtime PCR detection system (Bio-Rad, USA) and iQ^TM^SYBR^®^ Green supermix (Bio-Rad). For comparison of transcript levels between samples, a standard curve of cycle thresholds for several serial dilutions of a cDNA sample was established and then used to calculate the relative abundance of each gene. Values were then normalized to the relative amounts of *rPL7* cDNA. All PCR reactions were performed in duplicate.

### Immunofluorescence staining

To determine the presence and cell-type specific localization of PLA2G10 after P_4_ treatment, and during the estrous cycle and early pregnancy, uteri were fixed in 4% paraformaldehyde (PFA) and embedded in paraplast (Leica Biosystems, Germany). Uterine sections (5 µm) were deparaffinized, rehydrated, and subjected to antigen retrieval in 0.01 M sodium citrate buffer, pH 6.0, for 20 min. For immunofluorescence staining of ARG2 (Arginase 2), a marker for decidualization, frozen sections (13 µm) of IS on day 6 were fixed in 4% PFA, washed in PBS, and permeabilized with 0.1% triton-X 100 in PBS. Non-specific staining was blocked using Protein Block Serum-Free (Dako, Denmark) for 1 h. Then, sections were incubated overnight with primary rabbit-anti-PLA2G10 antibody (1:100, Santa Cruz Biotechnology, USA) for PLA2G10 or primary rabbit-anti-ARG2 antibody (1:200, abcam, USA) for ARG2 at 4 °C, washed in phosphate-buffered saline (PBS), and incubated with Alexa Fluor 488 goat-anti-rabbit secondary antibody (1:1000, Invitrogen Corp., USA) for 1 h at room temperature. Sections were washed in PBS, counterstained with propidium iodide (PI, Sigma-Aldrich) for 20 min, and mounted for observation using a LSM880 confocal microscope (Carl Zeiss, Germany).

### Hematoxylin & Eosin (H&E) staining and ALP activity assay

H&E staining and ALP activity assay were performed to evaluate gross histology of implanted embryos and decidualization in IS of the uterus with siPla2g10 on day 6, respectively. Frozen sections (13 µm) were fixed in 4% PFA, washed in PBS, and either stained with hematoxylin (Cancer Diagnostics, USA) and eosin (Richard Allan Scientific, USA) or incubated with a 100 mM Tris HCl buffer (pH 9.5) containing ALP substrate working solution (Vector Laboratories, SK-5400, USA). Slides were counterstained with fast red and mounted to observe ALP activity under light microscopy.

### Construction of expression and reporter vectors

A proximal region (− 840 to + 65) of *Pla2g10* promoter (p) was amplified from mouse genomic DNA by PCR with Forward 1 (5′-GCT AGC GGT GGT TCC AAG GTT TCA CTC AG-3′) and Reverse 1 (5′-CTC GAG GTC ACA GAG GTG GCC CAC AC-3′) primers. The amplified *Pla2g10*(p) was cloned into pGL4.10 vector (Promega) and named pGL4.10/Pla2g10(p)-840/ + 65. The vector was independently mutated at four PREs, namely − 801/− 794, − 356/− 349, − 310/− 303, and − 290/− 283 in *Pla2g10*(p)-840/ + 65 using the EZ change™ Site-directed Mutagenesis Kit (Enzynomics, Inc., Korea). The four mutated PREs were named pGL4.10/Pla2g10(p)-801mt, pGL4.10/Pla2g10(p)-356mt, pGL4.10/Pla2g10(p)-310mt, and pGL4.10/Pla2g10(p)-290mt, respectively. PRA and PRB cDNAs were provided by Dr. J.W. Jeong (Michigan State University, MI, USA). The cDNAs were cloned into a pcDNA3.1 *NheI*-*XhoI* site and named pcDNA3.1/PRA and pcDNA3.1/PRB, respectively.

### Transfection and luciferase assay

Ishikawa cells, human endometrial adenocarcinoma cells, were plated in 12-well plates with DMEM/F12 and 10% charcoal-stripped (CS)-FBS 24 h before transfection. pcDNA3.1, pcDNA3.1/PRA, or pcDNA3.1/PRB expression vectors were co-transfected with pGL4.10/Pla2g10(p)− 840/ + 65, pGL4.10/Pla2g10(p)-801mt, pGL4.10/Pla2g10(p)-356mt, pGL4.10/Pla2g10(p)-310mt, or pGL4.10/Pla2g10(p)-290mt vectors, and a pRL-null vector that was used as an internal control for normalization by GenePORTER^®^3000 Transfection Reagent (Genlantis, USA). The medium was replaced with DMEM/F12 and 2% CS-FBS with 1 µM P_4_ (Sigma-Aldrich) 4 h after transfection. Cells were harvested and analyzed for firefly and renilla luciferase activities using the Dual-Glo™ Luciferase Assay System (Promega) 24 h after transfection. Luminescence was measured with Synergy Mx™ (Bio Tek, Inc., USA).

### In vivo RNA interference of *Pla2g10* in mouse uterus

Knock-down of *Pla2g10* in mouse uterus was performed as previously described by Ruan et al. with some modifications [48]. Briefly, 100 pmol siPla2g10 (BIONEER Corp., Korea; 5′-GAA CAA AUG CCA AGA ACU U-3′) or siNC (BIONEER Corp.) were combined with 5 µl of lipofectamine 2000 in 10 µl of Opti-MEM. The solutions were injected into each uterine horn at 18:00—20:00 h on day 3 for in vivo RNA interference of *Pla2g10* in mouse uterus.

### Statistics

All values represent the mean ± standard deviation. The unpaired Student’s *t*-test was used for statistical evaluation. A p-value of less than 0.05 was considered statistically significant.

## Data Availability

All data generated or analyzed during this study are included in this published article.
